# Ni,Fe,Co-LDH Coated Porous Transport Layers for Zero-Gap Alkaline Water Electrolyzers

**DOI:** 10.3390/nano14050407

**Published:** 2024-02-23

**Authors:** Andrea Zaffora, Bartolomeo Megna, Barbara Seminara, Francesco Di Franco, Monica Santamaria

**Affiliations:** Department of Engineering, Palermo University, 90128 Palermo, Italy; bartolomeo.megna@unipa.it (B.M.); barbara.seminara@community.unipa.it (B.S.); francesco.difranco@unipa.it (F.D.F.); monica.santamaria@unipa.it (M.S.)

**Keywords:** oxygen evolution reaction, water splitting, layered double hydroxide, porous transport layer, zero-gap, stainless steel, electrode stability

## Abstract

Next-generation alkaline water electrolyzers will be based on zero-gap configuration to further reduce costs related to technology and to improve performance. Here, anodic porous transport layers (PTLs) for zero-gap alkaline electrolysis are prepared through a facile one-step electrodeposition of Ni,Fe,Co-based layered double hydroxides (LDH) on 304 stainless steel (SS) meshes. Electrodeposited LDH structures are characterized using Scanning Electron Microscopy (SEM) confirming the formation of high surface area catalytic layers. Finally, bi and trimetallic LDH-based PTLs are tested as electrodes for oxygen evolution reaction (OER) in 1 M KOH solution. The best electrodes are based on FeCo LDH, reaching 10 mA cm^−2^ with an overpotential value of 300 mV. These PTLs are also tested with a chronopotentiometric measurement carried out for 100 h at 50 mA cm^−2^, showing outstanding durability without signs of electrocatalytic activity degradation.

## 1. Introduction

Electrochemical water splitting is one of the most studied and promising ways to produce high purity green hydrogen, which could become the main energy carrier for the next future society [1]. In electrochemical water splitting, electrical energy is delivered to the system to electrochemically produce gaseous hydrogen and oxygen at the cathode and at the anode, respectively [2]. Nevertheless, cell efficiency can be low mostly because of the sluggish kinetics of the oxygen evolution reaction (OER), which is a four-electron process with a complex reaction mechanism. Therefore, finding highly active, stable, and low-cost materials for OER is still an existing challenge [3,4,5,6]. Usually, IrO_2_ is the electrocatalyst used for OER in Proton Exchange Membrane Water Electrolyzers (PEMWEs), leading to relatively low overpotential values, but it is expensive and Ir is a Critical Raw Material (CRM), hindering a worldwide deployment of water electrolysis. One of the strategies to avoid the usage of noble metals as electrocatalysts for OER is working in an alkaline environment [7]. Indeed, Alkaline Water Electrolyzers (AWEs) employ an aqueous alkaline solution, generally KOH-based with concentration 1–7 M at 40–90 °C, using a diaphragm or an anion exchange membrane (AEM) to separate the cathode and anode [8]. In the past, AWEs used gap-cells, i.e., there was a gap between the electrode and the cell separator, leading to a reduced efficiency, mostly at high current density where produced gases can produce a non-conducting layer over the surface of the electrodes. For this reason, gap-based AWEs are not capable to produce high pressure hydrogen, and the materials’ stability can be an important issue [8,9]. Zero-gap configuration leads to higher efficiency, and it foresees the usage of a so-called porous transport layer (PTL), which ensures electrical continuity between the catalytic layer and the current collector, and that is crucial to remove bubbles from the catalytic layer [10,11]. Functionalizing PTLs with a catalytic layer can be a way to further increase the cell efficiency [12,13].

Recently, Layered Double Hydroxides (LDHs) have been proposed as suitable electrocatalysts for OER, because of their activity and, most of all, because of their intrinsic stability in an alkaline environment [14,15]. LDHs can be prepared through several synthesis techniques, e.g., hydrothermal process, sol-gel, and urea hydrolysis [16,17,18,19], but usually these techniques are time-consuming, complex, and difficult to be scaled at industrial level. Electrochemical techniques, whether they can be coupled to renewable energy, can represent sustainable methods to produce materials at lab scale as well as at the industrial level because of their intrinsic scalability. In particular, electrodeposition is a mature technology, being used at industrial level for preparing high-throughput and large-scale functional coatings for many applications, that can be also carried out with a continuous roll-to-roll process further reducing manufacturing costs. Therefore, electrodeposition can be efficiently used to synthesize LDHs to be used to catalyze OER in alkaline environment [20,21,22].

Among all the possible LDHs, NiFe and FeCo LDHs are among the most active electrocatalysts for OER, and are used for industrial water electrolysis [2,23]. For this reason, here a facile and scalable electrodeposition process is used to functionalize 304 stainless steel meshes with Ni,Fe,Co-containing LDH to prepare PTLs for zero-gap-design AWEs. The prepared electrodes were studied using Scanning Electron Microscopy (SEM) and Raman spectroscopy, and then characterized with electrochemical techniques to assess their electrocatalytic activity toward OER in 1 M KOH aqueous solution. Durability of LDH/SS PTL electrodes was studied under harsh conditions at 50 mA cm^−2^ for 100 h to demonstrate their suitability to be used as electrodes for zero-gap AWEs.

## 2. Materials and Methods

### 2.1. Ni,Fe,Co-LDH Synthesis

Electrodes for OER were prepared using a one-step electrodeposition process carried out in a three-electrode cell. The substrate was a 304 AISI SS mesh, i.e., the effective PTL, pretreated before the electrodeposition with a chemical etching carried out in an aqueous solution 0.5 M H_2_SO_4_ for 10 min and then rinsed in acetone and milliQ water [12]. The LDH electrodeposition was carried out using FeSO_4_⸱7H_2_O, Ni(NO_3_)_2_⸱6H_2_O, Co(NO_3_)_2_⸱6H_2_O salts with a concentration of 0.1 M in water as solvent, solution pH = 3. For bi-metallic LDH, only two salts were used, whilst for the tri-metallic LDH, all the salts were used during the electrodeposition process. LDH synthesis was carried out by a potentiostatic deposition applying −1 V vs. Ag/AgCl/KCl sat., the latter used as a reference electrode, for 5 min. Two platinum nets with high surface areas were used as counter electrodes to have a uniform distribution of current density lines during the electrodeposition. All the samples were then thermally treated at 130 °C in air for 16 h.

### 2.2. PTLs Characterization

To study the morphological features of the electrodes, we used a FEI Quanta 200 FEG SEM (FEI Company, Hillsboro, OR, USA) coupled to an EDX (X-ray energy dispersive system) for compositional analysis. SEM micrographs were usually taken working at 30 kV without any metallization of the samples.

Raman spectra were acquired by means of a Renishaw InVia Raman Microscope (Renishaw, Wotton-under-Edge, UK), equipped with a 532 nm Laser and focused on the sample with a Leica MSDS microscope (Leica Microsystems, Wetzlar, Germany) using a 50x long working distance magnification lens. Maximum laser power was equal to 140 mW, and it was reduced by means of Holographic filters ranging from 1 to 10% according to the sample’s response, with an accumulation time equal to 10 s and averaged for four accumulations for each spectrum.

### 2.3. Electrochemical Characterization

OER performances of LDH-based electrodes were studied through Linear Sweep Voltammetries (LSVs) measurements carried out between 0.1 V and 1 V Hg/HgO/1 M KOH, the latter used as a reference electrode. The electrolyte was an aqueous solution 1 M KOH, and it was fed in a flow-through configuration to mimic the operating conditions of an AEMWE working with a zero-gap configuration. All the electrode potential, measured with respect to Hg/HgO reference electrode, were converted with respect to the Reversible Hydrogen Electrode (RHE) to make a reliable comparison with the literature data. The conversion of electrode potentials was done according to the following equation:

(1) $$E_{RHE}=E_{Hg/HgO}+0.1\;V+0.059\;pH$$

An iR compensation of 95% was applied to all the LSVs shown below. EIS spectra were recorded at several electrode potential to study the behavior of the electrodes in different operating conditions. Impedance spectra were then fitted using ZSimpWin software with equivalent electrical circuits described below.

### 2.4. Stability Test

Stability tests were carried out through galvanostatic measurements, with the same three-electrode configuration as for the electrochemical measurements, applying a current density of 50 mA cm^−2^ for 100 h in an aqueous solution 1 M KOH. Every 25 h, a stability test was stopped to record the LSV and impedance spectra to evaluate the electrocatalytic activity of the electrode.

## 3. Results and Discussion

### 3.1. Ni,Fe,Co-LDH Electrodeposition

To prepare functionalized PTLs with LDH structures, we carried out cathodic electrodeposition using SS mesh as substrate. LDH electrodeposition mechanism foresees, as a first step, the local increase of pH close to the substrate surface due to the reduction reaction of nitrate ions:

NO_3_^−^ + H_2_O + 2e^−^ → NO_2_^−^ + 2OH^−^ (2)

The equilibrium potential of reaction (2) is 0.63 V SHE considering electrodeposition conditions [24], we thus applied −1 V Ag/AgCl to drive nitrate ions reduction. Once OH^−^ ions are generated, bi or tri-metallic hydroxide precipitation occurs at the electrode surface to form an LDH structure as soon as the solubility product of the metal oxides is locally reached, according to the following reactions [21,25]:

M^x+^ + N^y+^ + (x + y)OH^−^ → MN(OH)_x+y_ (3)

or

M^x+^ + N^y+^ + Q^z+^ + (x + y + z)OH^−^ → MNQ(OH)_x+y+z_ (4)

where M^x+^, N^y+^, and Q^z+^ can be Fe^2+^, Ni^2+^, and Co^2+^ ions, in our case. It is noteworthy to mention that, during electrodeposition, at the applied electrode potential, other reduction processes can occur, i.e., water and metal ions reduction. The former can occur, leading to a pH increase:

2H_2_O + 2e^−^ → H_2_ + 2OH^−^ (5)

being the equilibrium potential of reaction (5) E_eq_ = −0.18 V SHE. Metal cations can be reduced to the zero-oxidation state, since the applied potential is negative with respect to the corresponding equilibrium potential [24].

### 3.2. LDH Morphological Characterization

**Figure 1:**
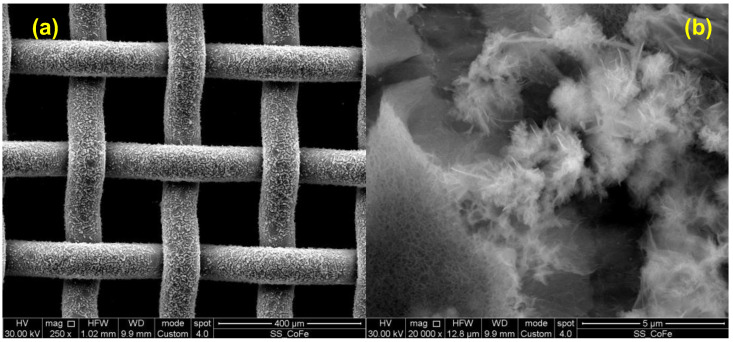
FeCo LDH micrographs taken with SEM at (**a**) 250x and (**b**) 20,000x magnification.

**Figure 2:**
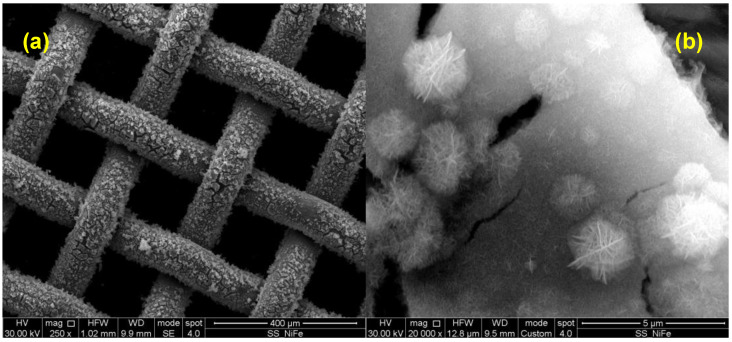
NiFe LDH micrographs taken with SEM at (**a**) 250x and (**b**) 20,000x magnification.

Morphologies of bi-metallic LDH layers, i.e., FeCo and NiFe LDHs, are reported in Figure 1 and Figure 2, respectively.

Bi-metallic LDHs present nanosheets morphology, which is a common feature of the LDH layers [21,22,25,26]. This morphology is very suitable for electrocatalytic application since it provides very high surface area, therefore exhibiting a high number of active sites for OER. Moreover, the space between the nanosheets can provide a path for evolved oxygen once it is produced, reducing eventual voltage drop due to a difficult bubble removal, decreasing the available surface area hindering the mass transport. It is worth noting that both FeCo-LDH and NiFe-LDH layers are uniform along the SS mesh substrate.

**Figure 3:**
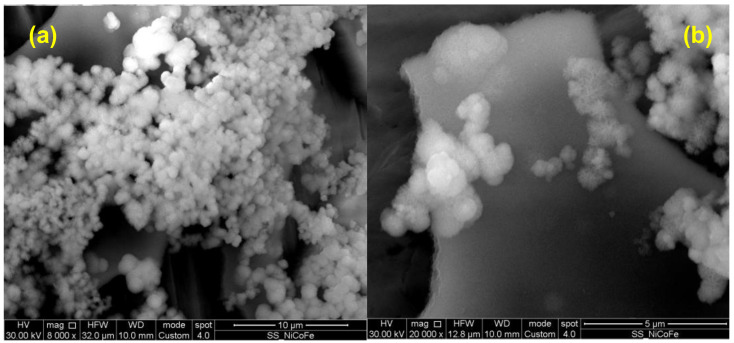
NiFeCo LDH SEM images taken at (**a**) 8000x and (**b**) 20,000x magnification.

Figure 3 shows the morphology of NiFeCo-LDH synthesized using electrodeposition. Nanosheets morphology is preserved also in tri-metallic LDH [27,28,29,30,31], but nanosheets have lower dimension (see Figure 3b) and are organized in clusters/nanospheres, instead forming a compact layer, as in the case of bi-metallic LDHs.

In Table 1, we report the compositional data derived from the EDX analysis.

However, data derived from the EDX analysis cannot be considered directly related to the composition of the LDH layer since the SS substrate contains both Fe and Ni.

**Figure 4:**
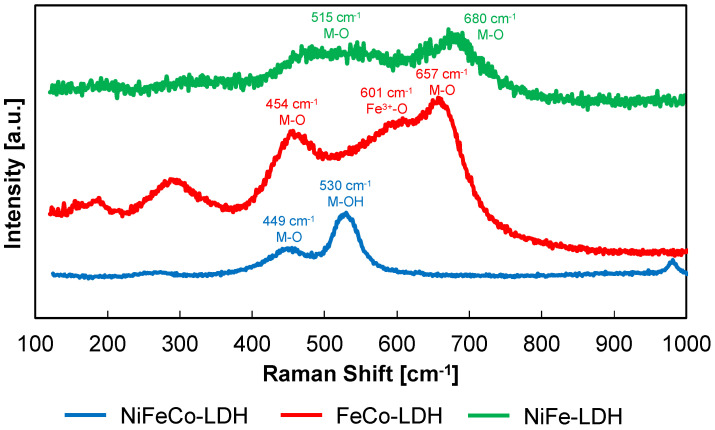
Raman spectra of electrodeposited NiFe, FeCo, and NiFeCo LDHs.

In Figure 4, we report the Raman spectra related to bi and tri-metallic LDHs studied in this work.

The Raman spectrum of NiFeCo LDH shows peaks at 449 cm^−1^ and 530 cm^−1^ associated with stretching vibrations of Co(OH)_2_ and Ni-OH, particularly the first to M-O and the second to M-OH stretching, where M can be Co, Ni, and Fe, while the peak at 970 cm^−1^ can be attributed to both the presence of some sulphate ion, deriving from the electrodeposition bath, or to the stretching of the Ni(OH)_2_ [32].

The spectrum of FeCo LDH shows broad peaks at 290, 454, 601, and 657 cm^−1^. The main peak at 657 cm^−1^ can be attributed to the symmetric stretching of M-O tetrahedral bonds, while the one at 601 cm^−1^ can be assigned to Fe^3+^-O stretching, and the one at 454 cm^−1^ be attributed to the asymmetric stretching of M-O bonds [33].

Finally, the spectrum of NiFe LDH only shows two broad peaks, at around 680 and 515 cm^−1^, that can be attributed, respectively, to symmetric and asymmetric vibration M-O bonds, considering the low quality of the spectrum.

### 3.3. OER Performance of Electrodeposited PTLs

**Figure 5:**
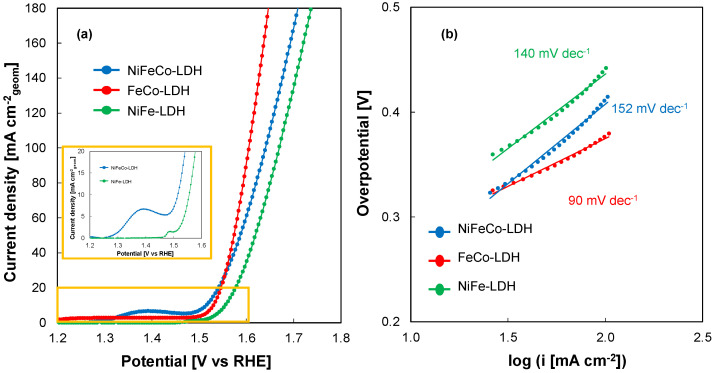
(**a**) LSVs recorded in 1 M KOH electrolyte of NiFe, FeCo, and NiFeCo-LDH-based electrodes in flow-through configuration. (**b**) Tafel plots derived from LSVs in (**a**).

Electrochemically prepared LDH-based PTLs were then characterized as OER electrodes in 1 M aqueous solution in a flow-through cell, i.e., electrolyte flows across the PTLs mimicking the real operating conditions of a PTL in a zero-gap electrolyzer. Figure 5a shows LSVs recorded for all the electrodes at 10 mV s^−1^.

As disclosed in Figure 5a, for FeCo LDH-based PTL, current density is very close to zero before the onset of O_2_ evolution, due to the presence of only non-faradaic processes. Whilst, in the case of Ni-containing LDH electrodes, an oxidation peak is present in the LSVs (see inset of Figure 5a) because of the oxidation of Ni^2+^ to Ni^3+^ (i.e., from Ni(OH)_2_ to NiOOH) in alkaline environment [15,25,34,35]. As reported by Louie and Bell [34], the composition of the electrocatalytic layer deeply influences the Ni oxidation peak, and in particular the Fe content in NiFe-LDH electrodes. When Fe content increases, the peak current density decreases, and the peak potential is more anodic with respect to those recorded with pure Ni electrode. This is because, for a pure Fe electrode, dissolution of Fe oxide/hydroxide almost coincides with the onset of OER, not showing any oxidation peak in LSV measurement. In the case of NiFeCo, Fe content is lower and Co presence adds a little contribution to the oxidation current because of Co^2+^/Co^3+^ oxidation [36], leading to a higher current density and a shift of oxidation peak toward cathodic direction. Overpotential corresponding to the onset of a current due to OER is also called onset overpotential, η_onset_, namely the difference between the measured electrode potential and the equilibrium potential of OER (i.e., 1.23 V RHE). The value of the onset overpotential is similar for all the electrodes, i.e., 250 mV, regardless of the bi or tri-metallic nature of LDH in the electrocatalytic layer. To compare the performance of the different prepared electrodes, the overpotential measured at 10 mA cm^−2^ was estimated as a function of the catalytic layer composition [37]. Using NiFeCo-LDH-based PTL, an overpotential of 290 mV is required to reach 10 mA cm^−2^, while 300 and 330 mV are required to reach the same current density using FeCo-LDH and NiFe-LDH-based electrodes, respectively. These overpotential values are comparable or slightly higher than those recorded for other d-metal-based LDHs [21], but they are obtained using a cheaper SS mesh substrate with respect to the usual expensive Ni foam, and a facile electrodeposition process that could be easily scaled to an industrial level.

Tafel slope is another important parameter for the evaluation of the electrocatalytic performance of a material. This value also gives information about the reaction mechanism related to a specific material for a specific reaction. Tafel plots related to the LSVs discussed before are reported in Figure 5b. First, the lowest Tafel slope is that related to the FeCo LDH-based electrode, i.e., 90 mV dec^−1^. This result implies that working with high current density values using a FeCo LDH-based electrode will require a lower overpotential, as it is possible to notice from the LSV curves shown in Figure 5a. Therefore, looking at the plots reported in Figure 5, the highest OER performances were assessed for the FeCo LDH-based electrode, reaching 50 and 100 mA cm^−2^ by applying 346 and 380 mV as overpotential values, respectively.

**Figure 6:**
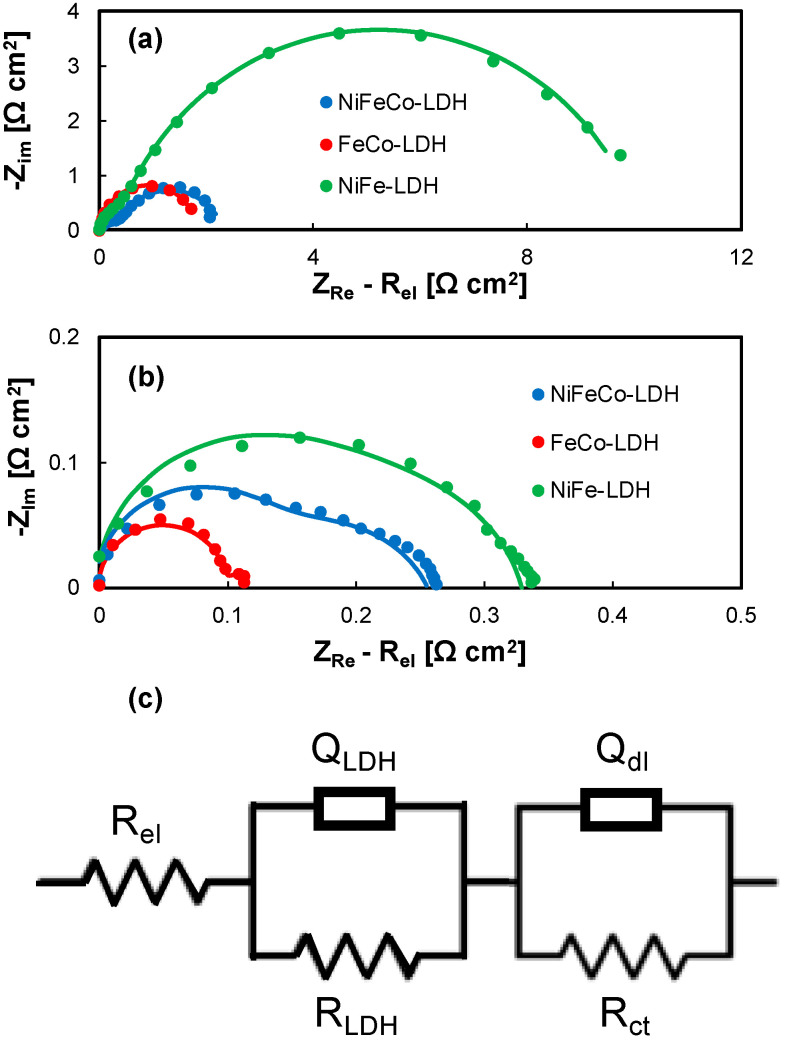
(**a**) Impedance spectra recorded at 1.53 V RHE and (**b**) impedance spectra recorded at 1.68 V RHE in 1 M KOH electrolyte of NiFe, FeCo, and NiFeCo-LDH-based electrodes in flow-through configuration. (**c**) Equivalent electrical circuit used for fitting EIS spectra.

To have more information about the electrocatalytic activity of LDH-based electrodes, EIS-based investigation was carried out, recording impedance spectra at two different electrode potentials, i.e., 1.53 V RHE and 1.68 V RHE, in 1 M KOH aqueous solution [38,39]. Nyquist plots of the recorded impedance spectra are shown in Figure 6a,b for all the investigated electrodes. The overall impedance, regardless of the studied electrode, significantly decreases the increasing anodic overpotential, i.e., from 1.53 to 1.68 V RHE, suggesting that, at 1.68 V RHE, OER is fully activated (see Figure 5a). To get more quantitative information about OER kinetics related to every electrode, impedance spectra were fitted using ZSimpWin software, where a suitable equivalent electrical circuit can be used to model the electrochemical behavior of the LDH/SS mesh system. From Nyquist plots shown in Figure 6a,b, the presence of two time constants can be assessed; therefore, the proposed equivalent circuit (see Figure 6c) consists of the electrolyte resistance, R_el_, in series with two parallel (RQ), where R is a resistance and Q is a Constant Phase Element (CPE) [40]. The impedance of a CPE can be expressed as [41]:

(6) $$Z_{CPE}=\frac{1}{{\left(j\omega \right)}^{n}Q}$$

where *n* is a frequency-independent parameter. When *n* = 1, Q has units of a capacitance, whilst when *n* = 0, Q has units of 1/(Ω cm^2^). Generally speaking, when *n* ≠ 1, system behavior can be attributed to surface inhomogeneity or to a distribution of time constants related to charge transfer reactions. One of the parallel accounts for the non-ideal double layer capacitance, Q_dl_, of the electrode, and for the charge transfer resistance, R_ct_, of the OER. R_ct_ provides information about the electrocatalytic activity of the electrode, since it is inversely proportional to the exchange current density of the process and the overpotential. The other (RQ) parallel accounts for the presence of LDH layers that can contribute to the measured impedance due to their electrical resistance (i.e., R_LDH_) and with non-ideal capacitance modelled with a CPE, Q_LDH_ [42]. Therefore, it is expected that R_ct_ strongly depends on potential, while R_LDH_ is not significantly affected by the overpotential value. Fitting parameters are reported in Table 2 and Table 3.

In agreement with the LSVs, at 1.53 V RHE, the reaction is not activated, thus the main contribution to the overall impedance arises from R_ct_, the highest being measured with the Co-free electrodes. Notably, using Brug formula to calculate double layer capacitance (C_dl_) from Q_dl_ [42,43], the highest C_dl_ values were assessed for a FeCo LDH-based layer (≅0.2 F cm^−2^). This result is very important because the C_dl_ value is directly related to the Electrochemical Active Surface Area (ECSA) leading to enhanced OER performance. At a higher overpotential (i.e., at 1.68 V RHE), R_ct_ is one order of magnitude lower. Conversely, R_LDH_ and Q_LDH_ are almost independent of potential, thus confirming that this contribution arises from the LDH layer, as above suggested, and in agreement with previous results reported in the literature [42]. Notably, the lowest R_LDH_ is measured for the FeCo-containing layer, i.e., the LDH with the best electrocatalytic activity. This experimental finding suggests that the FeCo-based catalyst is active toward oxygen evolution, but it is also more conductive with respect to the other layers, probably due to a lower thickness and/or to a lower hydroxide resistivity. These results are in agreement with the literature, where the crucial role of Fe in enhancing Ni and Co-based hydroxide activity is reported [44,45,46]. Moreover, a substrate of LDH-based electrocatalytic layer is SS, and it has been demonstrated that stainless steel can represent a good electrocatalyst for OER due to its capacity of forming active (oxy)hydroxides under oxidizing conditions [2,13].

### 3.4. Stability Test

**Figure 7:**
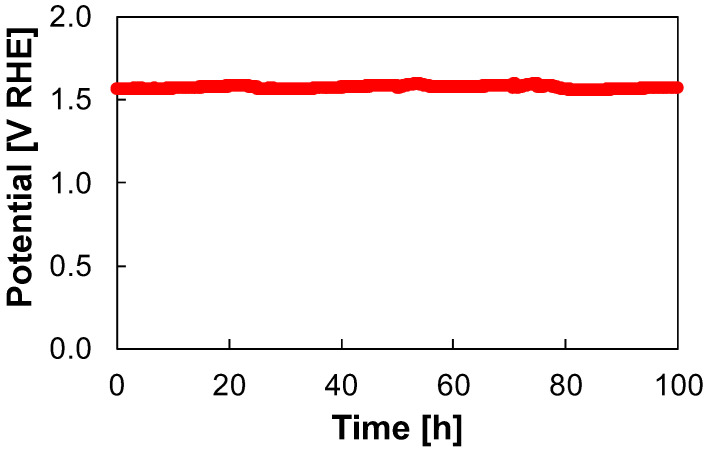
Potential vs. time curve related to the stability test for FeCo-based electrode carried out at 50 mA cm^−2^ for 100 h in 1 M KOH aqueous solution.

A good electrode for water electrolysis also needs a high stability in alkaline environment under strongly anodic polarization. To evaluate the electrocatalytic stability of the best electrode, i.e., the FeCo LDH-based one, a long-term galvanostatic measurement at 50 mA cm^−2^ was carried out for 100 h in 1 M KOH aqueous solution. The stability test curve is reported in Figure 7.

The overpotential is almost constant for (at least) 100 h during the stability test, with a value of 346 ± 20 mV. That is an outstanding result considering the harsh conditions of the stability test (i.e., current density and electrolyte composition). Moreover, the overpotential is comparable or even better with respect to many of the data reported in the literature for the stability test of LDH-based electrodes for OER in alkaline conditions that are summarized in Table 4. As a final remark, it is also important to stress that the stability test was stopped after 100 h without any failure.

**Figure 8:**
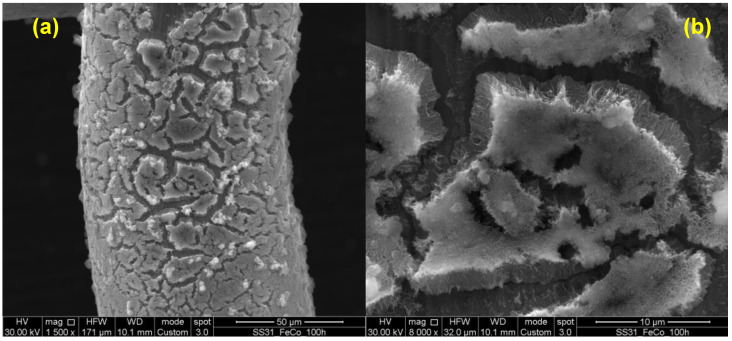
SEM images of FeCo LDH-based electrode after stability test at (**a**) 1500x and (**b**) 8000x magnification.

The stability of the FeCo LDH-based electrode was also studied with a morphological characterization by using electron microscopy. The SEM image acquired after 100 h stability test is shown in Figure 8.

As it is possible to notice, despite the fact that the coating is more cracked with respect to the sample before 100 h galvanostatic measurement (see Figure 1), nanosheets morphology is still preserved, in agreement with preserved electrocatalytic activity, as shown by the stability test results. The high stability of our FeCo LDH-based electrode can be due to the peculiar behavior of Fe. In fact, recently, Chung et al. [55] suggested that Fe can dissolve and re-deposit over hydroxides clusters, generating continuously stable Fe active sites. We also believe that this mechanism can be aided also by the presence of 304 stainless steel as a substrate of our FeCo LDH-based electrode, producing very active material even when the LDH layer dissolves during long-term measurements.

## 4. Conclusions

Facile and scalable electrodeposition processes were used to prepare functionalized 304 stainless steel porous transport layers for oxygen evolution reactions to be used in zero-gap alkaline water electrolyzers. Electrocatalytic layer was composed of bi and tri-metallic LDH composed of Ni, Fe, and Co. SEM investigation allowed us to assess a nanosheet-like morphology, as typical of LDH, prone to efficiently produce gaseous oxygen due to a very porous structure, rich of active sites for the water oxidation.

Prepared LDH-based electrodes exhibited excellent performance toward OER, being FeCo LDH-based PTL, the best electrode reaching 10 mA cm^−2^ with an overpotential value of 300 mV and a Tafel slope of 90 mV dec^−1^. This performance is due to the low charge transfer resistance, assessed using electrochemical impedance spectroscopy, related to the composition of the layer and to the highest electrochemical active surface area between the investigated electrodes.

The FeCo-LDH electrode also exhibited a high durability, withstanding a 100 h stability test carried out at 50 mA cm^−2^ in 1 M KOH aqueous solution without any sign of degradation of electrocatalytic activity, still preserving nanosheet-like morphology. This study provides a green, facile, and scalable strategy to design highly active and stable electrocatalyst-coated porous transport layers for next-generation zero gap alkaline water electrolyzers.

## Figures and Tables

**Table 1:** Atomic composition of all the LDH-based electrodes obtained by EDX.

Sample	O [at.%]	S [at.%]	Cr [at.%]	Fe [at.%]	Ni [at.%]	Co [at.%]
NiFe-LDH	47.6	2.1	8.4	34.4	7.6	-
FeCo-LDH	53.4	3.0	6.5	27.2	2.4	7.5
NiFeCo-LDH	50.0	2.5	7.4	28.7	6.0	5.6

**Table 2:** Fitting parameters related to the spectra recorded at 1.53 V RHE (Figure 6a).

Sample	R_el_ [Ω cm^2^]	R_LDH_ [Ω cm^2^]	Q_LDH_ [S s^n^ cm^−2^]	*n*	R_ct_ [Ω cm^2^]	Q_dl_ [S s^n^ cm^−2^]	*n*
NiFe-LDH	0.26	0.33	2.1 × 10^−3^	1	9.86	0.012	0.81
FeCo-LDH	0.24	0.01	4.5 × 10^−3^	0.9	1.82	0.21	0.93
NiFeCo-LDH	0.32	0.34	8.5 × 10^−3^	0.87	2	0.13	0.80

**Table 3:** Fitting parameters related to the spectra recorded at 1.68 V RHE (Figure 6b).

Sample	R_el_ [Ω cm^2^]	R_LDH_ [Ω cm^2^]	Q_LDH_ [S s^n^ cm^−2^]	*n*	R_ct_ [Ω cm^2^]	Q_dl_ [S s^n^ cm^−2^]	*n*
NiFe-LDH	0.26	0.18	2.7 × 10^−3^	1	0.16	0.04	0.83
FeCo-LDH	0.24	0.02	5.0 × 10^−3^	0.93	0.10	0.23	1
NiFeCo-LDH	0.32	0.13	4.1 × 10^−3^	1	0.13	0.21	0.73

**Table 4:** Stability performance of LDH-based electrodes for OER in alkaline conditions. n.s.: not specified.

LDH	Synthesis Method	Test Duration [h]	Current Density [mA cm^−2^]	Electrolyte	Overpotential [mV]	Reference
FeCo	Electrodeposition	50	10	1 M KOH	260	[26]
3D NiFe	Electrodeposition	2	10	1 M KOH	260	[47]
NiFe, NiCo NiCu, CuCo FeCo, FeCu	Electrodeposition	3 0.3	10 100	0.1 M KOH	n.s.	[22]
NiFe	Hydrothermal	95	100	1 M KOH	240	[48]
NiCo	Electrodeposition	36	13	1 M KOH	270	[49]
Ru-doped NiFe	Hydrothermal	6	10	1 M KOH	240	[50]
NiFeW	Solvothermal	6	10	1 M KOH	270	[51]
Rh-NiFe	Hydrothermal	7	10	1 M KOH	210	[52]
NiFe LDH@NiCoP	Hydrothermal	100	10	1 M KOH	230	[53]
NiFe-rGO	Precipitation	10	10	1 M KOH	240	[54]
FeCo	Electrodeposition	100	50	1 M KOH	346	This work

## Data Availability

Research data are available upon request.
